# Characterization of the complete chloroplast genome of *Lonicera tangutica* (Caprifoliaceae), an ornamental and medicinal plant in China

**DOI:** 10.1080/23802359.2022.2054376

**Published:** 2022-03-28

**Authors:** Xiaoyu Wang, Jing He, Xueping Su, Bixiang Xu, Yan Liu, Yanhong Tang, Shengnan Sun, Ping Li, Chengzhou Zhao

**Affiliations:** a Hunan Tianjin Pharmaceutical Co., Ltd., Changsha, China; b College of Horticulture, Hunan Agricultural University, Changsha, China; c Tibetan Medicine Research Center, Qinghai University, Xining, China

**Keywords:** Chloroplast genome, *Lonicera tangutica*, evolution and genetic diversity, Leguminosae

## Abstract

In the present study, the complete chloroplast genome of *Lonicera tangutica* is presented and characterized for the first time. The complete chloroplast genome was 156,121 bp in length, including 23,899 bp inverted repeat (IR) regions, an 89,466 bp large single-copy (LSC) region, and an 18,851 bp small single-copy (SSC) region. A total of 129 genes, including 37 tRNA genes, eight rRNA genes, and 84 protein-coding genes, were annotated, and the overall GC content of the chloroplast genome was 38.35%. Two introns in the *ycf3* gene and a single intron in another gene were detected. Maximum-likelihood phylogenetic analysis indicated that *L. tangutica* has a very close evolutionary relationship with *Lonicera praeflorens*, *Lonicera hispida*, *Lonicera fragrantissima*, and *Lonicera stephanocarpa*. These results are valuable for studying the evolution and genetic diversity of *L. tangutica.*

*Lonicera tangutica* Maxim. (Maximowicz, 1878, *L. tangutica*) belongs to the Caprifoliaceae family and mainly occurs in forests, hillside grasslands, and streamside shrubs at an altitude of 1600–3500 m in Shaanxi, Ningxia, Gansu, Qinghai, Hubei, Sichuan, Yunnan, and Tibet (Committee for the Pharmacopoeia of PR China 2015). *Lonicera* species have been used as traditional medicine as they exhibit antiallergic, anti-inflammatory, antibacterial, and antiviral properties based on many active ingredients, including caffeoylquinic acid, cerebrosides, nitrogen-containing iridoid glycosides, and triterpene glycosides (Peng et al. 2000; Lin et al. 2008; Zheng et al. 2012; Yu et al. 2015; Kong et al. 2017). The root and root bark of *L. tangutica* are used to treat carbuncle; its branches can be used to treat asthma, boils, and carbuncle after peeling, and its flower buds can absorb heat, detoxify, and prevent malaria. Although *L. tangutica* has high medicinal value, research on its genetic resources has not yet been reported. In the present study, we report the complete chloroplast genome of *L. tangutica* and provide intraspecies phylogenetic relationships. The assembled and determined chloroplast genome sequence of *L. tangutica* will be a useful resource for future genetic and genomic research.

Young and healthy leaf samples were collected from Mengda Tianchi in Xining city, Qinghai province, China (102°36′ E, 35°42′ N) for total genomic DNA extraction. A specimen was deposited at the Tibetan Medicine Research Center of Qinghai University (https://www.qhu.edu.cn/, Chengzhou Zhao, qhdxzcz2016@163.com) under the voucher number TMSGS21000. The improved cetyltrimethylammonium bromide (CTAB) method was used to extract the total genomic DNA (Doyle and Doyle 1987), and the extracted DNA was sequenced with the Illumina Hiseq X Ten Sequencing System (Illumina, San Diego, CA) by Biomarker Biotech Co., Ltd. (Beijing, China). The filtered reads were assembled using the programs SPAdes and Getorganelle with its congener *Lonicera japonica* chloroplast genome (GenBank accession number: NC_026839.1) as the initial reference genome (Jin et al. 2020). The chloroplast genome sequence was annotated through the online program CPGAVAS 2, followed by manual correction (Shi et al. 2019). Finally, the validated complete chloroplast genome sequence was deposited in GenBank under the accession number MZ962399 (Figure 1).

**Figure 1.**
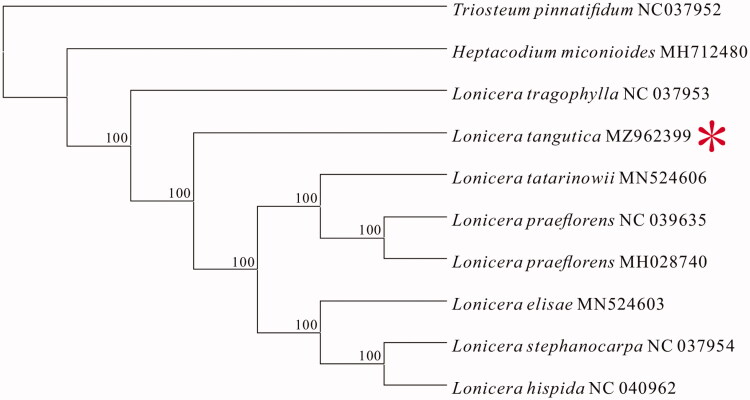
Consensus maximum-likelihood (ML) tree based on the complete chloroplast genome of *L. tangutica* and nine other species of the Caprifoliaceae family.

The complete chloroplast genome was 156,121 bp in length, consisting of a pair of inverted repeat (IR) regions of 23,899 bp each, a large single-copy region of 89,466 bp, and a small single-copy region of 18,851 bp. A total of 129 genes were annotated, including 37 tRNA genes, eight rRNA genes, and 84 protein-coding genes. The overall GC content of the cp genome was 38.35%. Furthermore, except for the *ycf3* gene that harbors two introns, all other genes, including seven PCG genes (*rps16*, *atpF*, *rpoC1*, *rpl16*, *ndhA*, *ndhB*, and *rpl2*), two other genes (*petB* and *petD*), and five tRNA genes (*trnA*-UGC, *trnI*-GAU, *trnG*-UCC, *trnL*-UAA, and *trnV*-UAC), possess only a single intron.

To reveal the phylogenetic position of *L. tangutica* within the Caprifoliaceae family, we constructed an evolutionary tree with nine other species from the Caprifoliaceae family and *Lonicera tangutica* using the maximum-likelihood (ML) in MEGA 7.0 software with 1000 bootstrap replicates. Our result confirmed that *L. tangutica* is closely related to *Lonicera praeflorens*, *Lonicera hispida*, *Lonicera tatarinowii*, *Lonicera elisae*, and *Lonicera stephanocarpa*.

## Data Availability

The genome sequence data obtained from this study are openly available in GenBank of NCBI (https://www.ncbi.nlm.nih.gov/) under accession number MZ962399. The associated BioProject, SRA, and Bio-Sample numbers are PRJNA770114, SRR16327067, and SAMN22209141, respectively.
